# No Evidence for Retinal Damage Evolving from Reduced Retinal Blood Flow in Carotid Artery Disease

**DOI:** 10.1155/2015/604028

**Published:** 2015-10-19

**Authors:** Henning Heßler, Hanna Zimmermann, Timm Oberwahrenbrock, Ella Maria Kadas, Janine Mikolajczak, Alexander U. Brandt, Andreas Kauert, Friedemann Paul, Stephan J. Schreiber

**Affiliations:** ^1^NeuroCure Clinical Research Center, Charité-Universitätsmedizin Berlin, 10117 Berlin, Germany; ^2^Department of Neurology, Königin-Elisabeth-Herzberge Hospital, 10365 Berlin, Germany; ^3^Department of Neurology, Charité-Universitätsmedizin Berlin, 10117 Berlin, Germany

## Abstract

*Introduction*. Carotid artery disease (CAD) comprising high-grade internal carotid artery stenosis (CAS) or carotid artery occlusion (CAO) may lead to ipsilateral impaired cerebral blood flow and reduced retinal blood supply. *Objective*. To examine the influence of chronic CAD on retinal blood flow, retinal morphology, and visual function. *Methods*. Patients with unilateral CAS ≥ 50% (ECST criteria) or CAO were grouped according to the grade of the stenosis and to the flow direction of the ophthalmic artery (OA). Retinal perfusion was measured by transorbital duplex ultrasound, assessing central retinal artery (CRA) blood flow velocities. In addition, optic nerve and optic nerve sheath diameter were measured. Optical coherence tomography (OCT) was performed to study retinal morphology. Visual function was assessed using high- and low-contrast visual paradigms. *Results*. Twenty-seven patients were enrolled. Eyes with CAS ≥ 80%/CAO and retrograde OA blood flow showed a significant reduction in CRA peak systolic velocity (no-CAD side: 0.130 ± 0.035 m/s, CAS/CAO side: 0.098 ± 0.028; *p* = 0.005; *n* = 12). OCT, optic nerve thicknesses, and visual functional parameters did not show a significant difference. *Conclusion*. Despite assessable hemodynamic effects, chronic high-grade CAD does not lead to gaugeable morphological or functional changes of the retina.

## 1. Introduction

Carotid artery disease (CAD) comprising high-grade carotid artery stenosis (CAS) or carotid artery occlusion (CAO) of the internal carotid artery (ICA) was shown to be associated with restricted ocular blood flow as measured by Doppler sonography [1–5]. The higher the grade of CAS, the more relevant the impairment of the retinal blood flow, especially if the ophthalmic artery (OA) shows a retrograde blood flow [6]. A common carotid artery occlusion (CCA-O) impedes the blood flow of the ipsilateral internal and external carotid artery (ECA) and is another risk factor for limited orbital perfusion [7, 8]. Ocular manifestations of CAD are a common finding, for instance, occurring as amaurosis fugax caused by acute embolic central retinal artery occlusion or as a chronic retinal ischemia in the form of ocular ischemic syndrome (OIS). Retinal examination in CAD patients is generally performed in direct time relation to the occurrence of clinical symptoms like sudden or progressive visual loss. However, it remains unclear whether subclinical retinal abnormalities or visual impairment occurs. Ultrasound allows reliable dynamic assessment of orbital blood flow, measured as central retinal artery (CRA) flow velocity. It can also be used for structural analysis of optic nerve sheath diameter (ONSD) and is a proven diagnostic tool to evaluate papilledema in intracranial pressure with comparable results to MRI examinations [9]. Optical coherence tomography (OCT) allows reliable quantification of retinal layers and is able to detect retinal axonal and neuronal loss even in the absence of clinical visual symptoms in multiple sclerosis and other CNS diseases [10–12]. In this pilot study, we analyze the effects of CAD-induced chronic reduced retinal blood flow on optic nerve thickness and retinal morphology and function.

## 2. Methods

### 2.1. Patients

Patients were recruited from two hospital ultrasound laboratories (Departments of Neurology: Charité-Universitätsmedizin Berlin and Königin-Elisabeth-Herzberge Hospital, Berlin) and an outpatient practice in Berlin between August 2011 and May 2012. After database screening for chronic high-grade ICA stenosis and ICA occlusion, patients were selectively invited for an additional study appointment. Inclusion criteria were unilateral high-grade CAS ≥ 50% (ECST criteria) [13], CAO or CCA-O, and age > 18. Exclusion criteria were contralateral CAS ≥ 50% (ECST criteria), contralateral CAO or CCA-O, ICA stent or carotid endarterectomy (CEA), and any ophthalmological diseases potentially affecting the retina (i.e., glaucoma, advanced diabetes, and retinopathies). Patients with diabetes were included if they had not suffered from any ophthalmological symptoms in their medical history.

### 2.2. Patient Grouping

The cohort was classified into groups according to the following hemodynamic criteria:

 Group 1: patients with CAS 50% to 79%.
 Group 2: patients with CAS ≥ 80% or CAO with a nonretrograde OA.
 Group 3: patients with CAS ≥ 80% or CAO with a retrograde OA.
 Group 4: patients with CCA-O.

A missing blood flow in the OA was defined as nonretrograde blood flow.

### 2.3. Ethics Statement

The study was approved by the Institutional Review Board (IRB) of the Charité-Universitätsmedizin Berlin and was conducted in accordance with the Declaration of Helsinki in its current version and applicable German laws. All participants gave informed written consent.

### 2.4. Ultrasound

All patients underwent a complete ultrasound examination of the extra- and intracranial brain supplying arteries using a Toshiba PowerVision ultrasound system (SSA-370A, Toshiba, Japan). Extracranial flow velocities were measured with a linear probe (7.0 MHz), intracranial vessels, and the flow direction of the OA with a sector probe (1-2 MHz). Degree of ICA stenosis was determined according to the multiparametric DEGUM ultrasound criteria [14].

**Figure 1:**
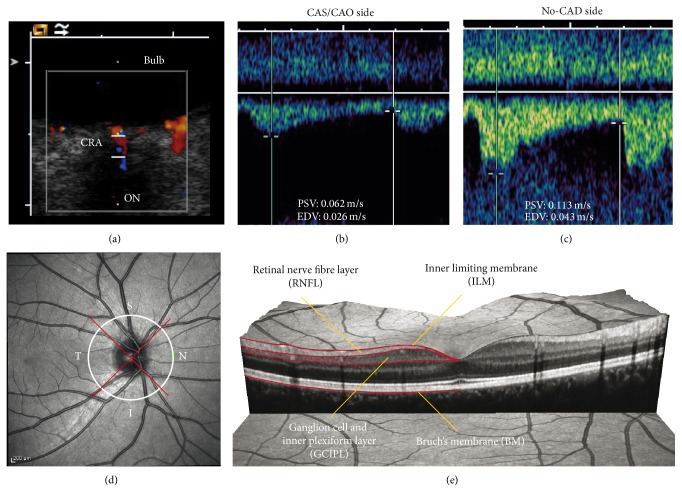
(a)–(c) Transorbital ultrasound measurement of flow velocities in the central retinal artery (CRA) in a patient with carotid artery occlusion (CAO) and retrograde OA blood flow. (a) Transorbital B-mode scan of the optic nerve and color-mode imaging of the CRA. (b) Flow velocities ipsilateral to the CAO. Besides the reduced absolute flow velocity, there is a markedly delayed systolic flow rise. (c) Flow velocity of the no-CAD side. Note the normal steep systolic flow rise. (d) Exemplary peripapillary OCT ring scan with sectors: temporal (T), superior (S), inferior (I), and nasal (N). (e) Exemplary presentation of an OCT macular volume scan with segmentation of the retinal nerve fiber layer (RNFL) and combined ganglion cell and inner plexiform layer (GCIPL).

Transorbital ultrasound was performed with the Siemens ACUSON (Sequoia 512, Siemens, Germany) and a linear probe (15.0 MHz). The mechanical index (MI) was reduced (MI < 0.24) according to current standards. As measures of retinal blood supply, the CRA peak systolic velocity (PSV), end diastolic velocity (EDV), and the resistive index (RI) were determined without use of angle correction (see, e.g., Figures 1(a)–1(c)). Measurements of the optic nerve sheath diameter (ONSD) and optic nerve diameter (OND) were performed according to the method of Helmke and Hansen [15, 16]. According to the examination conditions, single or multiple measurements were performed. In case of multiple measurements, the mean value was used for further analysis.

### 2.5. Optical Coherence Tomography

Retinal nerve fiber layer (RNFL) thickness, total macular volume (TMV), combined ganglion cell and inner plexiform layer (GCIPL) volume, and optic nerve head volume (ONHV) were obtained from retinal OCT scans (Heidelberg Spectralis, Heidelberg Engineering, Germany; Spectralis software version 5.3.3.0; Eye Explorer Software 1.6.4.0). RNFL thickness was measured with a 12° ring scan (3.4 mm diameter) around the optic nerve head using the device's standard protocol and segmentation algorithm with high resolution mode (1536 A-scans per B-scan) and activated true track (Figure 1(d)). Whenever possible, the maximum number of averaging frames in the automatic-real-time mode (ART) was used. Beside the global RNFL thickness, the superior, temporal, inferior, and nasal RNFL quadrant thicknesses were analyzed.

The macular volume was measured using a custom protocol which generates 61 B-scans focusing the fovea, at a scanning angle of 30° × 25° and a resolution of 768 A-scans per B-scan (ART = 13). The TMV was calculated by estimating the distance between the inner limiting membrane and Bruch's membrane in a 6 mm diameter cylinder using the Spectralis software segmentation algorithm.

The GCIPL was segmented from the macular scan with semiautomatic beta-software provided by the manufacturer (Eye Explorer Viewing Module Version 5.7). All scans were checked for algorithm errors and inaccurate segmentation lines were corrected manually (Figure 1(e)).

The ONHV was determined from a custom protocol scan which generates 145 B-scans focusing the optic nerve head at an angle of 15° × 15° and a resolution of 384 A-scans per B-scan (ART = 10). A custom Matlab algorithm was used to quantify ONHV [17, 18].

All scans were acquired by one experienced operator and were checked for pathologies and sufficient quality according to the OSCAR-IB criteria before undergoing quantitative analysis [19, 20].

### 2.6. Visual Function

Visual function was tested monocularly under best-corrected conditions by one trained examiner with the Functional Vision Analyzer (Stereo Optical Co., Chicago, Illinois) with a simulated distance of 20 ft. High-contrast visual acuity (HCVA) was tested with Early Treatment Diabetic Retinopathy Study (ETDRS) charts under photopic conditions (85 cd/m^2^). Low-contrast visual acuity (LCVA) was tested with the Functional Acuity Contrast Test (FACT) under photopic (85 cd/m^2^) and mesopic (3 cd/m^2^) conditions without glare [21]. LCVA was calculated as the area under the curve (AUC) as previously described [22–24].

## 3. Results

### 3.1. Patients

In total, 30 patients could be recruited. Out of these, 27 had unilateral CAS or a CAO occlusion, while three patients had a CCA occlusion. Because of the small sample size, results of the latter group were analyzed separately. A flow chart of the different patient cohorts in this study is shown in Supplementary Figure 1 in Supplementary Material available online at http://dx.doi.org/10.1155/2015/604028. Among the remaining 27 patients with CAD, eight patients had a CAS between 50 and 79%, seven a CAS ≥ 80% or CAO with a nonretrograde OA, and 12 a CAS ≥ 80% or CAO with a retrograde OA. Demographic details of the total cohort and the stenosis groups are shown in Table 1. All patients were clinically asymptomatic with no CAD-related symptoms in the last two months.

### 3.2. Flow Velocities in the Central Retinal Artery

**Figure 2:**
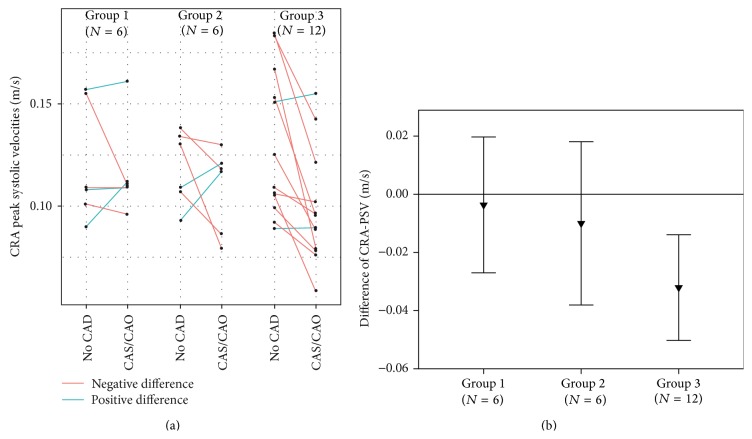
Results of peak systolic velocity (PSV) in the central retinal artery (CRA). (a) shows the individual measurements in stenosis subgroups. (b) shows PSV differences (PSV intereye difference = PSV CAS/CAO side − PSV no-CAD side) of stenosis subgroups. CAD: carotid artery disease; CAS: carotid artery stenosis; CAO: carotid artery occlusion, Group 1: CAS 50–79%; Group 2: CAS ≥ 80%/CAO with nonretrograde OA; Group 3: CAS ≥ 80%/CAO with retrograde OA.

Three patients had to be excluded because the CRA was not detectable on both sides. In the remaining cases, PSV was significantly reduced on the CAS/CAO side compared to the no-CAD side in the total cohort (*p* = 0.007) and in Group 3 (CAS ≥ 80%/CAO and retrograde OA, *p* = 0.005) (see Table 2). Side-to-side comparisons of the individual measures and intrapatient, intereye differences are shown in Figure 2. EDV and the RI showed no significant side-to-side differences in the total cohort or the subgroups (see Table 2). The level of PSV reduction in the CRA correlates significantly with the degree of CAS (*p* = 0.002, rho = −0.599, *N* = 24).

### 3.3. Subgroup CCA-Occlusion (CCA-O)

The three CCA-O patients showed the following results: PSV CCA-O side: 0.079 ± 0.022 m/s, no-CAD side: 0.147 ± 0.046 m/s, EDV CCA-O side: 0.030 ± 0.009 m/s, no-CAD side: 0.043 ± 0.015 m/s, and RI CCA-O side: 0.62 ± 0.07 m/s, no-CAD side: 0.71 ± 0.02 m/s. Due to the small group size (*N* = 3), no statistical tests were performed. All three patients had to be excluded from further morphological and functional analysis (one ocular bulb deformation, one study drop-out, and one pronounced epiretinal gliosis in a measurement-changing level).

### 3.4. Optic Nerve Thickness

There were no significant differences of the ON thickness parameters (OND, ONSD) in the total cohort between the CAS/CAO side and the no-CAD side. Detailed results of ON thickness measurements are given in Table 2. Group analyses showed a significant difference in Group 2 (CAS ≥ 80%/CAO, nonretrograde OA) between CAS/CAO side and no-CAD side of the OND (*p* = 0.027) and the ONSD (*p* = 0.028), while there were no significant side-to-side differences in Group 1 (CAS 50–79%) or Group 3 (CAS ≥ 80%/CAO, retrograde OA).

### 3.5. Optic Coherence Tomography

Eleven patients had to be excluded from OCT analysis due to potentially measurement influencing pathologies of the retina, among those five patients with maculopathies, two with local retina atrophies, and one with macular edema due to traction, or diseases which could affect the retinal morphology (two patients with amblyopia, one with optic neuritis in medical history). One patient did not complete the examinations due to an acute infection. Exemplary presentation of retinal pathologies with measurement-changing extent is shown in Supplementary Figure 2.

Fifteen patients were included into morphological and functional analysis of the retina. Due to the small sample size, no subgroup analyses were performed. Instead, correlation of OCT measures with the degree of CAS and the CRA-PSV was analyzed to evaluate the relationship between OCT parameters and the orbital hemodynamic situation. CRA-PSV was only available for 13 patients because the CRA could not be detected on both sides in two patients.

Analysis of global and quadrant RNFL thickness, TMV, GCIPL, and ONHV showed no significant differences between CAS/CAO side and no-CAD side (Table 3).

Furthermore, no significant correlation with the degree of CAS or the CRA-PSV was found (Spearman-Rho test, data not shown).

### 3.6. Visual Function

Fifteen patients were included into functional analysis of the retina. Analysis of HCVA, photopic LCVA, and mesopic LCVA showed no significant differences between CAS/CAO side and no-CAD side (Table 3). Furthermore, no significant correlation with the degree of CAS or the CRA-PSV was found (Spearman-Rho test, data not shown).

### 3.7. Correlation between Morphological and Clinical Parameters

**Figure 3:**
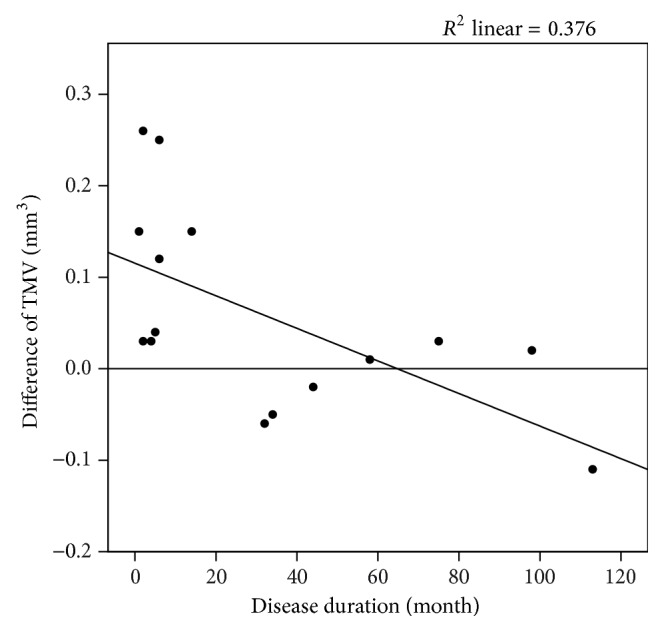
Scatterplot describing the significant correlation (*p* = 0.005) between the disease duration of the CAS/CAO and the intereye difference of the total macular volume (TMV). Patients with initial CAS/CAO diagnosis in the last 15 months show thickening of the TMV. TMV intereye difference = TMV CAS/CAO side − TMV no-CAD side. CAD: carotid artery disease; CAS: carotid artery stenosis; CAO: carotid artery occlusion. Spearman-Rho test was used.

All OCT parameters (RNFL thicknesses, TMV, GCIPL, and ONHV) were tested for correlations with ON thickness (OND, ONSD), patient age, disease duration of CAS, and functional visual parameters (HCVA, LCVA). There was a negative correlation between TMV difference (TMV difference = TMV CAS/CAO side − TMV no-CAD side) and disease duration (*p* = 0.005). The scatterplot indicates that there is a cluster of patients with a disease duration of <15 months, which has higher TMV (see Figure 3). All other correlation analyses showed no significant results. Furthermore, we grouped patients with CAD-associated symptoms (amaurosis fugax (AF), transient ischemic attack (TIA), or stroke) and analyzed OCT parameters. Seven patients of the OCT cohort (*N* = 15) had CAD-associated symptoms in their medical history (2 AF, 1 TIA, and 4 strokes). OCT parameters on CAS/CAO side with CAD-associated symptoms were compared with OCT parameters on CAS/CAO side without CAD-associated symptoms. No significant differences were found (nonparametric Mann-Whitney *U* test was used).

## 4. Discussion

This study confirms that chronic high-grade CAS/CAO in combination with a retrograde OA blood flow leads to a significant blood flow reduction in the ipsilateral CRA. Furthermore, our data suggest that these hemodynamic changes without a clinically manifest pathology do not result in changes in ON thickness (ONSD and OND), the retinal morphology (RNFL thickness, TMV, GCIPL volume, and ONHV), or functionality (HCVA, photopic and mesopic LCVA).

Kang et al. (2014) showed a subfoveal thinning of the choroid in three patients with high-grade CAS and OIS by enhanced depth imaging (EDI) OCT (Heidelberg Spectralis) [25]. In this study, we did not investigate the choroid thickness because our images were recorded without EDI mode. Furthermore, there are currently no reliable automatic segmentation methods available for the choroid. Instead, we analyzed state-of-the-art retinal OCT parameters, which were successfully applied to detect retinal neurodegeneration in MS and other neurodegenerative diseases, and established optic nerve ultrasound measures.

The significant side-to-side differences of ONSD (*p* = 0.028) and OND (*p* = 0.027) in Group 2 (CAS ≥ 80%/CAO, nonretrograde OA) seem not to be related to the reduced blood flow in the CRA. If this was the case, there should be a significant side-to-side difference of ON thicknesses as well in Group 3 (CAS ≥ 80%/CAO, retrograde OA), since we found significant PSV reduction in this group in contrast to PSV in Group 2 (see Table 2).

However, patients with initial CAS/CAO diagnosis in the last 15 months seem to show a thickening of the TMV on the CAS/CAO side compared to no-CAD side (Figure 3). That might be explained by an initial retinal edema, an excessive vascularization, or venous dilatation (overall diameter of retinal vessels is included in TMV) due to ischemia, which were regressive during time. Vascular changes and retinal edema are possible manifestations of an early stage of OIS [26, 27]. In contrast, RNFL thickness in the nasal quadrant tended to show a thinning on CAS/CAO side compared to no-CAD side; however, this difference was not significant.

This study shows that PSV reduction in the CRA correlates significantly with the degree of CAS (*p* = 0.002). Blood flow velocities in the downstream CRA in CAS ≥ 80% or CAO are lower if the OA flow is retrograde compared to those with a nonretrograde OA. To assess whether this was an effect relevant to the perfusion of the eye, additional parameters like pulse pressure or ocular perfusion pressure, assessed by, for example, oculoplethysmography, would have been desirable but were not assessed in our patient group. However, data from the literature in fact suggest that PSV in the CRA significantly correlates with the systolic pressure in the CRA and OA, supporting our hypothesis of impaired ocular circulation [3]. Results of the small CCA-O cohort support the hemodynamic findings and extent of blood flow reduction, which might occur in such a “worst case scenario.” In this constellation the ocular blood supply is severely impaired and collateral compensation can only be provided via anastomoses from the contralateral external carotid artery. Unfortunately, our cohort size was small and the drop-out rate did not allow analyzing retinal structure and visual function. Patients with such a specific condition tend to be clinically less stable which might have been the reason for the low recruitment numbers.

In our study we were not able to perform a full ophthalmologic examination including measurement of intraocular pressure. It is therefore possible that we missed mild glaucomatous changes that would have not been apparent in OCT. Spherical refraction was compensated by manually focusing on the fundus but spherical refraction was not assessed independently. Consequently we did not employ exclusion criteria, that is, in regard to high myopia. These are two clear limitations of our study. However, given the negative results (also in comparison to normative data in the device which makes clinical diagnosis of major confounding conditions improbable), we do not believe that influence on our results is relevant. The negative findings in morphological and functional analyses may also be explained with the small cohort size. To detect small changes, bigger cohort sizes might be necessary. The decision to choose the intrasubject comparisons (no-CAD side versus CAS/CAO side) instead of intersubject comparisons (CAS/CAO eyes versus eyes from healthy control subjects), which could have gained a larger cohort size, was made with respect to the comparability due to the comorbidities. Individual systemic factors have a greater comparability in intrasubject than in intersubject comparisons. Still, the lack of a healthy control cohort might be considered a weakness, because some patients might have had CAD < 50% on the no-CAD side.

The cohort in this study matches the expected demographic frame for CAD patients very well and is comparable to other CAD studies [1–5].

In total, twelve patients had to be excluded from the OCT analyses, mainly due to retinal pathologies in a measurement-changing extent. This finding exemplifies, on the one hand, that the applicability of quantitative OCT analysis is limited in elderly multimorbid patients. On the other hand, there might be more appropriate parameters as the ones chosen in this study. Concerning the brain, CAD more often causes embolic infarctions while hemodynamic patterns with only 5–8% of cases are relatively rare [28]. Xu et al. introduced the so-called RNFL defect, a localized sector in which the RNFL contour line touched or dipped into the <1% reference zone for a length of less than 180°, due to microinfarctions of the retina of which appearance correlates with arterial hypertension [29]. Wang et al. found RNFL defects in 48.1% (±4.0 SD, *N* = 154) of patients with acute and in 40.7% (±2.8 SD, *N* = 206) of patients with previous stroke [30]. In a retrospective analysis of our data no such RNFL defects according to definition of Xu et al. could be found, even though our cohort includes patients with arterial hypertension (66.7%) and previous stroke (33.3%). A reason for this finding could be an improper definition of RNFL defects, our small sample size, or different ethnic groups (Asian versus Caucasian).

## 5. Conclusion

High-grade CAS or CAO in combination with retrograde OA flow leads to a significant blood flow reduction in the CRA but not to morphological or functional changes of the retina. Our findings indicate that some patients with a recent CAD diagnosis can show a significant thickening of the ipsilateral TMV. We recommend further studies with a larger cohort size to evaluate the retinal changes in patients with recent CAD diagnosis or with acute embolic stroke and to develop and use adapted OCT parameters for the detection of retinal embolic defects.

## Supplementary Material

Supplementary Figure 1 shows a flowchart of the different patient cohorts.
Supplementary Figure 2 shows OCT macular thickness maps and cross-sectional images for examples of retinal pathologies with measurement-changing extent.

## Figures and Tables

**Table 1:** Demographic details of the total cohort and stenosis subgroups. SD: standard deviation; min: minimal; max: maximal; CAD: carotid artery disease comprising carotid artery stenosis (CAS) and carotid artery occlusion (CAO); HLP: hyperlipoproteinemia; PAD: peripheral artery disease; TIA: transient ischemic attack; Group 1: CAS 50–79%; Group 2: CAS ≥ 80% or CAO with nonretrograde OA; Group 3: CAS ≥ 80% or CAO with retrograde OA.

	Total cohort	Stenosis subgroups		
		Group 1	Group 2	Group 3
Patients *N*	27	8	7	12
Sex				
Male	17 (63%)	7 (87.5%)	2 (28.6%)	8 (66.7%)
Female	10 (37%)	1 (12.5%)	5 (71.4%)	4 (33.3%)
Age (years)				
Mean ± SD	61 ± 10	66 ± 4	62 ± 7	58 ± 12
(min–max)	(35–76)	(61–72)	(50–71)	(35–76)
Disease duration CAD (months)				
Mean ± SD	46 ± 50	21 ± 20	74 ± 55	46 ± 56
(min–max)	(1–177)	(2–58)	(6–177)	(1–169)
Unilateral CAD				
50–79%	8 (29.6%)	8 (100%)	0	0
80–99%	9 (33.3%)	0	5 (71.4%)	4 (33.3%)
Occlusion	10 (37.0%)	0	2 (28.6%)	8 (66.7%)
Comorbidity				
Art. hypertension	18 (66.7%)	7 (87.5%)	5 (71.4%)	6 (50.0%)
HLP	14 (51.9%)	4 (50.0%)	3 (42.9%)	7 (58.3%)
Coronary artery disease	8 (29.6%)	4 (50.0%)	1 (14.3%)	3 (25.0%)
Diabetes mellitus	4 (14.8%)	2 (25.0%)	1 (14.3%)	1 (8.3%)
PAD	3 (11.1%)	1 (12.5%)	1 (14.3%)	1 (8.3%)

**Table 2:** Side-to-side differences (no-CAD versus CAS/CAO) of sonographic results in the CRA peak systolic velocity (PSV), end diastolic velocity (EDV), and resistive index (RI) and the optic nerve thicknesses optic nerve sheath diameter (ONSD) and optic nerve diameter (OND). Significant findings in PSV for the total cohort (*p* = 0.007) and Group 3 (*p* = 0.005) and ONSD (*p* = 0.028) and OND (0.027) in Group 2. CAD: carotid artery disease; CAS: carotid stenosis; CAO: carotid artery occlusion, Group 1: CAS 50–79%; Group 2: CAS ≥ 80% or CAO with nonretrograde OA; Group 3: CAS ≥ 80% or CAO with retrograde OA; SD: standard deviation; CoV: coefficient of variation. Paired sample Wilcoxon sign rank test was used for intereye comparisons (no-CAD versus CAS/CAO).

		Total cohort	Stenosis groups		
			Group 1	Group 2	Group 3
PSV m/s ± SD (CoV)	*N*	24	6	6	12
	No-CAD	0.125 ± 0.030 (0.238)	0.120 ± 0.029 (0.239)	0.119 ± 0.018 (0.152)	0.130 ± 0.035 (0.272)
	CAS/CAO	0.105 ± 0.025 (0.241)	0.116 ± 0.023 (0.195)	0.109 ± 0.021 (0.191)	0.098 ± 0.028 (0.287)
	*p* value	**0.007**	0.893	0.463	**0.005**

EDV m/s ± SD (CoV)	*N*	24	6	6	12
	No-CAD	0.037 ± 0.017 (0.460)	0.034 ± 0.010 (0.308)	0.032 ± 0.009 (0.273)	0.040 ± 0.022 (0.544)
	CAS/CAO	0.034 ± 0.014 (0.413)	0.038 ± 0.016 (0.434)	0.033 ± 0.008 (0.225)	0.032 ± 0.016 (0.491)
	*p* value	0.493	0.463	0.917	0.147

RI ±SD (CoV)	*N*	24	6	6	12
	No-CAD	0.72 ± 0.08 (0.109)	0.71 ± 0.06 (0.089)	0.73 ± 0.04 (0.054)	0.71 ± 0.10 (0.141)
	CAS/CAO	0.69 ± 0.10 (0.145)	0.68 ± 0.12 (0.176)	0.70 ± 0.04 (0.052)	0.68 ± 0.12 (0.170)
	*p* value	0.063	0.416	0.093	0.307

ONSD cm ± SD (CoV)	*N*	27	8	7	12
	No-CAD	0.477 ± 0.060 (0.125)	0.473 ± 0.066 (0.140)	0.468 ± 0.045 (0.095)	0.484 ± 0.066 (0.113)
	CAS/CAO	0.480 ± 0.066 (0.138)	0.465 ± 0.096 (0.207)	0.500 ± 0.047 (0.093)	0.478 ± 0.054 (0.113)
	*p* value	0.471	1.000	**0.028**	0.937

OND cm ± SD (CoV)	*N*	27	8	7	12
	No-CAD	0.291 ± 0.045 (0.156)	0.287 ± 0.059 (0.207)	0.270 ± 0.037 (0.137)	0.306 ± 0.037 (0.119)
	CAS/CAO	0.300 ± 0.042 (0.141)	0.295 ± 0.059 (0.199)	0.304 ± 0.027 (0.089)	0.300 ± 0.040 (0.134)
	*p* value	0.275	0.726	**0.027**	0.929

**Table 3:** Side-to-side differences (no-CAD versus CAS/CAO) of OCT and functional measurements with no significant findings. RNFL: peripapillary retinal nerve fiber layer; G: global; S: superior; N: nasal; I: inferior; T: temporal; TMV: total macula volume; GCIPL: ganglion cell/inner plexiform layer; ONHV: optic nerve head volume; HCVA: high-contrast visual acuity; LCVA: low-contrast visual acuity (photopic = 85 cd/m^2^, mesopic = 3 cd/m^2^); CAD: carotid artery disease; CAS: carotid artery stenosis; CAO: carotid artery occlusion, ± standard deviation. Paired sample Wilcoxon sign rank test was used for intereye comparisons (no-CAD versus CAS/CAO).

	*N*	No-CAD	CAS/CAO	*p* value
RNFL-G thickness (*µ*m)	15	101 ± 8	99 ± 9	0.363
RNFL-S thickness (*µ*m)	15	126 ± 11	126 ± 14	0.363
RNFL-N thickness (*µ*m)	15	76 ± 10	73 ± 14	0.100
RNFL-I thickness (*µ*m)	15	128 ± 15	129 ± 21	0.977
RNFL-T thickness (*µ*m)	15	70 ± 11	69 ± 11	0.637
TMV (mm^3^)	15	8.62 ± 0.41	8.68 ± 0.41	0.083
GCIPL volume (mm^3^)	15	1.96 ± 0.12	1.97 ± 0.13	0.344
ONHV (mm^3^)	13	1.253 ± 0.158	1.275 ± 0.288	0.972
HCVA (decimal)	15	1.11 ± 0.45	1.01 ± 0.31	0.206
LCVA photopic (AUC)	14	1.925 ± 0.198	1.997 ± 0.097	0.158
LCVA mesopic (AUC)	12	1.676 ± 0.333	1.698 ± 0.186	0.937
