# Supplementary material for: No Evidence for Retinal Damage Evolving from Reduced Retinal Blood Flow in Carotid Artery Disease
Source: Biomed Res Int. 2015 Oct 19;2015:604028. doi: 10.1155/2015/604028 (PMC4628976; doi:10.1155/2015/604028)
Supplement: Supplementary file 1 — Supplementary Figure 1 shows a flowchart of the different patient cohorts. Supplementary Figure 2 shows OCT macular thickness maps and cross-sectional images for examples of retinal pathologies with measurement-changing extent. [file 604028.f1.pdf]

No Evidence for Retinal Damage Evolving from Reduced Retinal Blood Flow in Carotid Artery Disease

Henning Heßler[1], Hanna Zimmermann[1], Timm Oberwahrenbrock[1], Ella Maria Kadas[1], Janine Mikolajczak[1], Alexander U. Brandt[1], Andreas Kauert[2], Friedemann Paul[1, 3], Stephan J. Schreiber[3]

1) NeuroCure Clinical Research Center, Charité - Universitätsmedizin Berlin, Germany

2) Department of Neurology, Evangelisches Krankenhaus Königin Elisabeth Herzberge Berlin, Germany

3) Department of Neurology, Charité - Universitätsmedizin Berlin, Germany

Supplementary Figures

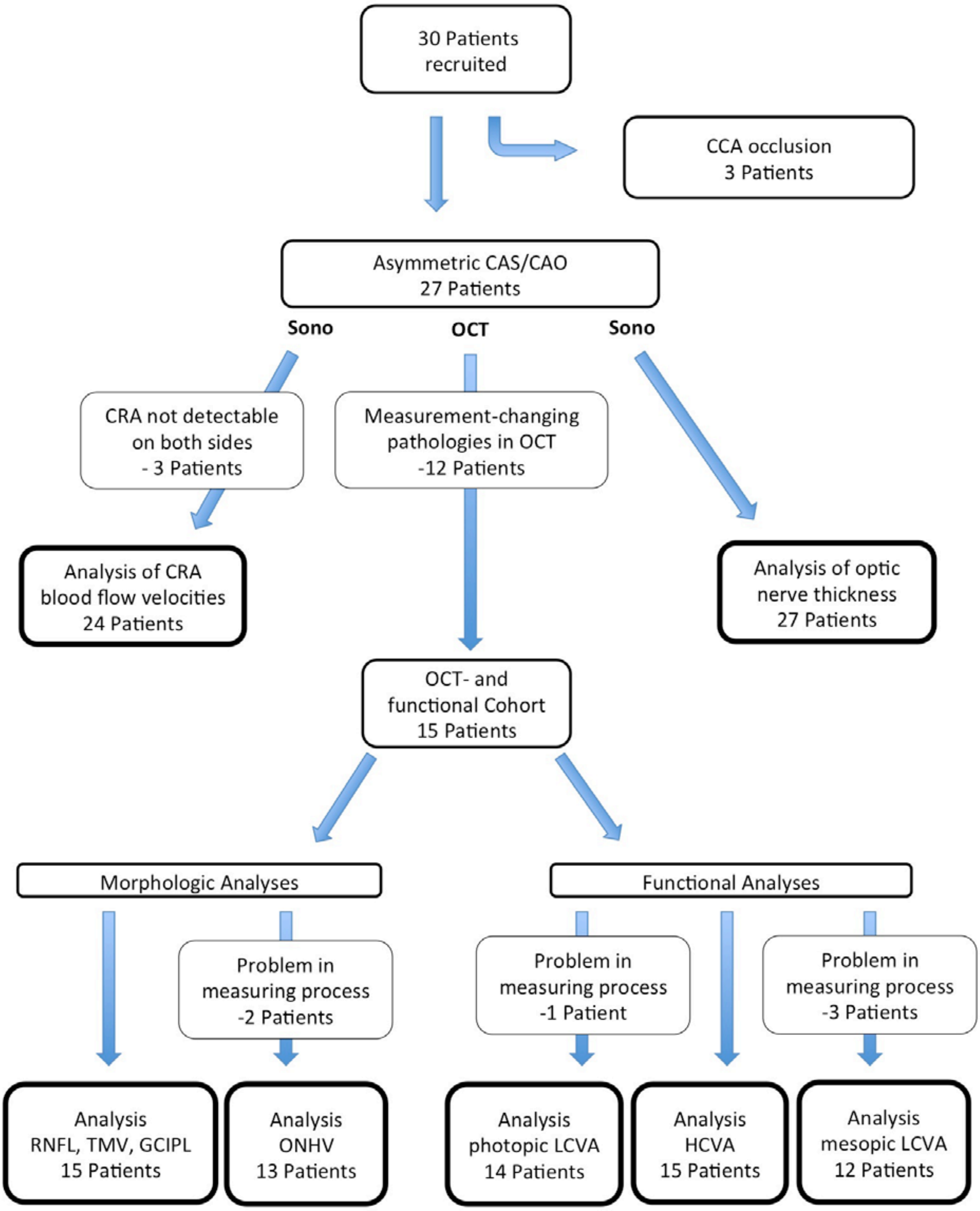

Supplementary-Figure 1: Flowchart of different patient cohorts. *CAD* carotid artery disease, *CAS* carotid artery stenosis, *CAO* carotid artery occlusion, *CCA* common carotid artery, *CRA* central retinal artery, *OCT* optical coherence tomography, *RNFL* global retinal nerve fiber layer thickness, *TMV* total macular volume, *GCIPL* ganglion cell / inner plexiform layer, *ONHV* optic nerve head volume, *HCVA* high contrast visual acuity, *LCVA* low contrast visual acuity (photopic=85 cd/m^2, mesopic=3 cd/m^2).

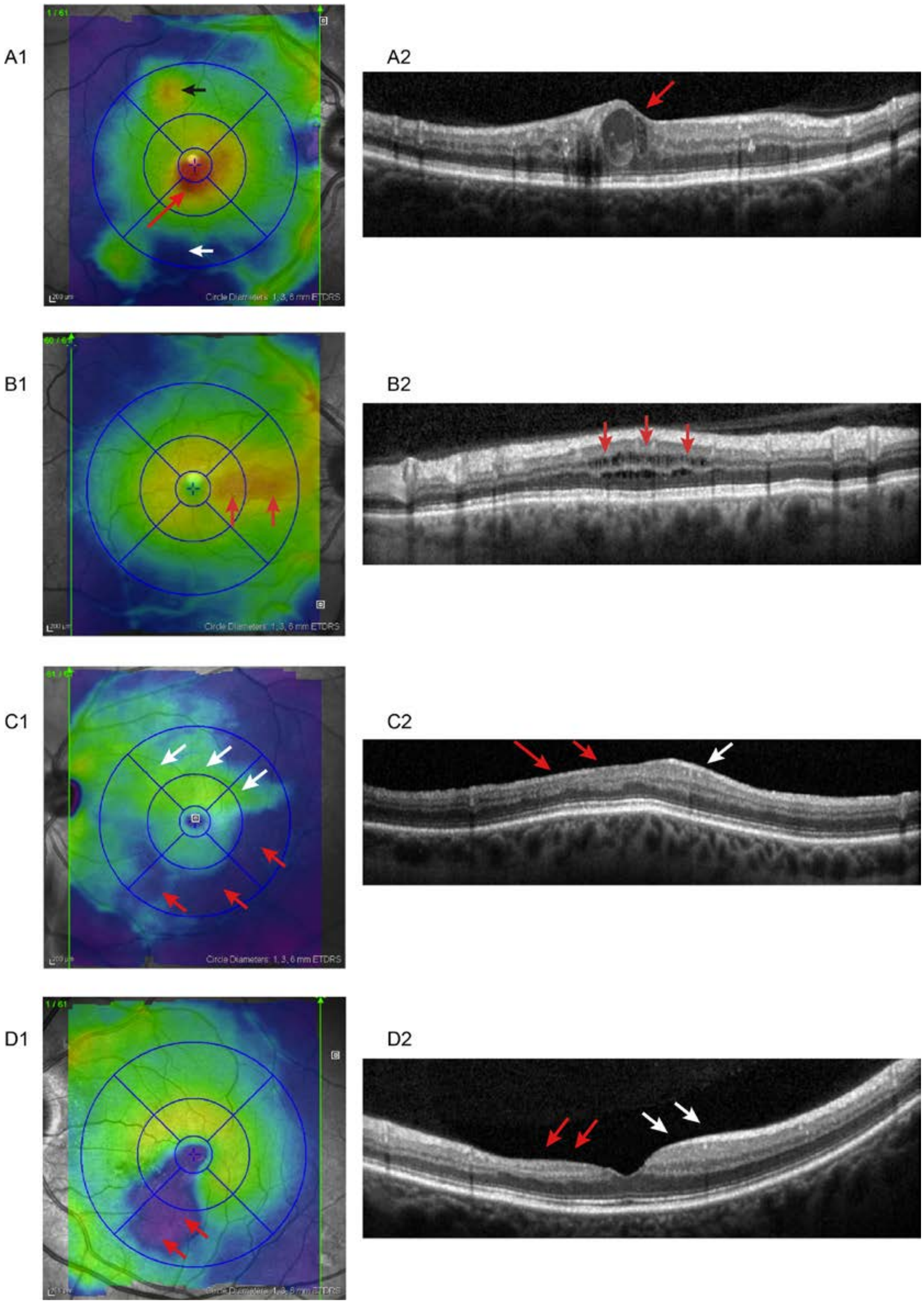

Supplementary-Figure 2: OCT macular thickness maps and cross-sectional images of retinal pathologies with measurement-changing extent. Patient **A** showed unknown diabetic macular edema on both sides. **A1** presents local thickenings (black arrow) and local thinning (white arrow) next to pronounced macular edema (red arrow in **A1** and **A2**), which can be seen in the corresponding B-Scan **A2**. Patient **B** presented a pronounced intraretinal edema resulting in a vitreoretinal traction-syndrome. **B1** shows a strung out thickening (red arrows) in the area between macula and papilla, **B2** shows the corresponding B-Scan. The retina seems lifted, an intraretinal edema has occurred (red arrows). Patient **C** presented a focal atrophy on the CAS/CAO side, which is compatible to a retinal artery branch occlusion. **C1** shows a thinning in the inferior retinal quadrants (red arrows) in comparison to the normal configuration of the retinal thickness (white arrows). **C2** the corresponding B-scan shows a thinning of

the upper retinal layers (red arrows) compared to the normal retinal configuration (white arrow). Patient **D** presented a focal atrophy on the no-CAD side. **D1** shows the well-defined atrophic area (red arrows), **D2** the corresponding B-Scan: the upper retinal layers are atrophic (red arrows) compared to the normal retinal configuration.
